# Creation of identical multiple focal spots with prescribed axial distribution

**DOI:** 10.1038/srep14673

**Published:** 2015-10-01

**Authors:** Yanzhong Yu, Qiwen Zhan

**Affiliations:** 1 College of Physics & Information Engineering, Quanzhou Normal University, Quanzhou, Fujian 362000, China; 2 Electro-Optics Program, University of Dayton, 300 College Park, Dayton, Ohio 45469, USA

## Abstract

We present a scheme for the construction of coaxially equidistant multiple focal spots with identical intensity profiles for each individual focus and a predetermined number and spacing. To achieve this, the radiation field from an antenna is reversed and then gathered by high numerical aperture objective lenses. Radiation patterns from three types of line sources, i.e., the electric current, magnetic current and electromagnetic current distributions, with cosine-squared taper are respectively employed to generate predominately longitudinally polarized bright spots, azimuthally polarized doughnuts, and focal spots with a perfect spherically symmetric intensity distribution. The required illuminations at the pupil plane of a 4Pi focusing configuration for the creation of these identical multiple focal spots can be easily derived by solving the inverse problem of the antenna radiation field. These unique focal field distributions may find potential applications in laser direct writing and optical microscopy, as well as multiple-particle trapping, alignment, and acceleration along the optical axis.

Over the last decade, the study of cylindrical vector (CV) beams, including the radially polarized beam, azimuthally polarized beam, and generalized CV beam, especially when focused under a high numerical aperture (NA) lens, has drawn much attention^1^,^2^,^3^,^4^ owing to their fascinating properties and intriguing applications, including optical microlithography^5^, microscopy^6^, spectroscopy^7^, and so on. Through modulating the incident CV beam with specific filters in a high NA focusing system, a variety of peculiar optical field distributions in the focal region have been engineered, e.g., optical needle^8^,^9^, optical chain^10^,^11^, optical bubble or cage^4^,^12^, spherical spot^13^, and multiple spots^14^,^15^,^16^,^17^,^18^,^19^,^20^, etc. However, the creation of double or multiple identical focal spots along the optical axis with prescribed characteristics has received much less attention compared with that of a single spot^14^. The formation of double focal spots was reported in^14^,^15^ by using a modulated radial-variant vector field and a concentric multi-belt pure phase filter, respectively. In 2010, Yan *et al.*^16^ utilized a spherical wave expansion technique to create multiple spherical spots in a 4Pi focusing system with a radially polarized beam. Liu *et al.*^17^ reported equidistant multiple spots by modulating a radially polarized beam with an amplitude apodization filter in 2013. Subsequently, the generation of isotropic focal spots in a 4Pi scheme was suggested in^18^ through selecting the proper position of an annular aperture and optimizing its size under the condition of equal transverse and longitudinal spot sizes. Recently, the Zhan group^19^ produced two identical spherical spots by reversing and focusing the radiation pattern emitted from a dipole antenna with the length of an odd integer number of a half wavelength. Furthermore, a systematic method for generating multiple focal spots with controllable polarization and intensity distribution through focusing the radiation pattern from an optimized electric dipole array under a high NA lens was proposed^20^.

However, applying the previously reported methods to create bi-focus or multi-focus with identical intensity profiles for each focal spot, especially with controllable number and spacing, are very complicated and inflexible. In the present paper, we propose a simple yet flexible approach for generating coaxially equidistant multiple spots with identical intensity profiles for each focal spot and variable number and interval. This is achieved in a 4Pi configuration by reversing and focusing the field radiated from a line source antenna with length L and amplitude distribution modulated by a cosine-squared taper along its extent. The generated three-dimensional identical focal spots are uniformly distributed within the range of –*L*/2 ~ +*L*/2 along the optical axis. The number of focal spots *N* and the interval between two adjacent spots are readily controllable by the parameters *L* and *N*. The engineered multiple focal spots may find potential applications in the multiple-particle trapping and manipulation^21^, delivery and self-assembly^22^,^23^.

## Results

### Proposed scheme

**Figure 1:**
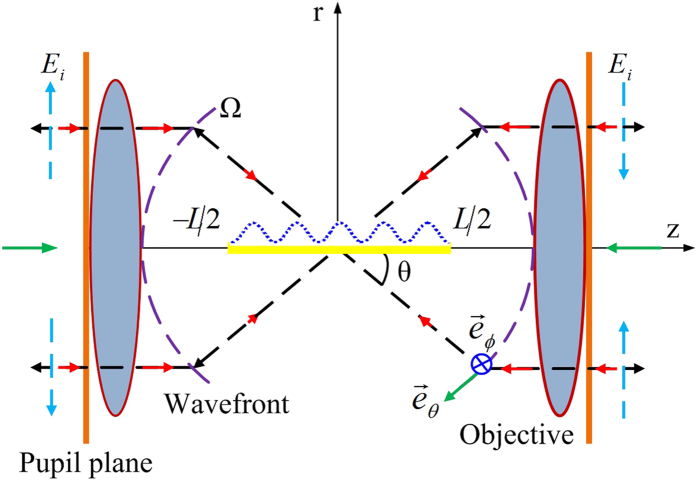
Schematic diagram of a 4Pi focusing system consisting of two confocal high-NA objective lenses. A line antenna (denoted by solid black line) with a cosine-squared taper (denoted by dashed blue curve) centered at the foci of two high-NA objectives is aligned along the optical axis. The radiation field from the line antenna (denoted by black arrows) is collected and inversely propagated to the focus region (denoted by red arrows). *ê*_*θ*_ and *ê*_*ϕ*_ are perpendicular to each other on Ω.

**Figure 2:**
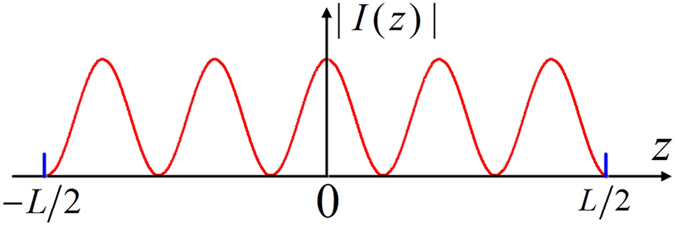
Amplitude distribution of an electric current line source with a cosine-squared taper (*N* = 5).

The method of reversing and focusing a radiation field from an antenna to create a specific desired focal field has been well developed by the Zhan group^24^,^25^,^26^. The design principle and procedure in a 4Pi focusing system are described in detail in^19^,^27^,^28^ and therefore will not be covered in this paper. In this work, we introduce a tapered line source antenna, which is often used in the antenna engineering field. A line antenna for which the electric current distribution is a cosine-squared taper along its extent is adopted here. Assuming that, in the 4Pi configuration, a z-directed tapered line source is centered symmetrically on the origin and along the z-axis (see Fig. 1), the electric current distribution is given by^29^


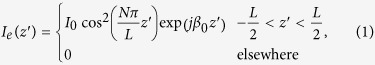


where *I*_*0*_ is a constant; *L* represents the length of the tapered line source; *N* = 1, 2, …, is an integer; and *β*_*0*_ denotes the phase shift per unit length along the line source. An example of this current amplitude distribution |*I*_*e*_(*z*)| = |cos^2^(*Nπz*/*L*)| for *N* = 5 is illustrated in Fig. 2. The far-zone electric field obtained by a radiation integral over the current distribution can be expressed as^29^


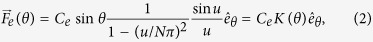


where


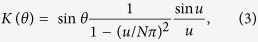


and *u* = *L*(*β* cos *θ* + *β*_*0*_)/2. *β* and *θ* denote the wave number and radiation angle between the radiation direction and z-axis, respectively. *C*_*e*_ is a constant related to electric current *I*_*e*_, and *ê*_*θ*_ is a unit vector of the radiation field. If the electric current distribution on the tapered line source is replaced by the magnetic current, then, according to the principle of electromagnetic duality, one can obtain the far-zone electric field expression for the magnetic current line source as


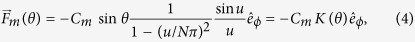


where *C*_*m*_ is a constant related to magnetic current *I*_*m*_ and *ê*_*ϕ*_ is a unit vector along the azimuthal direction. From Eq. (3) and/or Eq. (4), in conjunction with the Richards-Wolf theory^30^,^31^, we can derive the incident field expressions at the pupil plane of the high NA aplanatic lens obeying the sine condition as


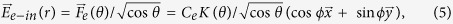



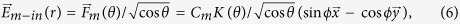



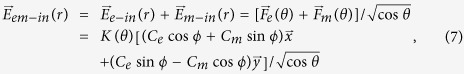


where 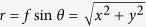, *f* is the focal length of the objective lens, and 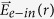 and 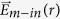 are the required incident fields evaluated by the patterns radiated solely from the electric current and magnetic current line sources, respectively. 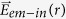 denotes the pupil field with the combined radiation from the electric current and magnetic current line sources. Consequently, the electric field distributions in the neighborhood of the focus can be computed by the Richards–Wolf vectorial diffraction method^30^,^31^ as


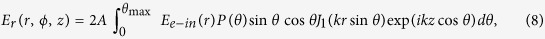



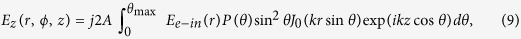



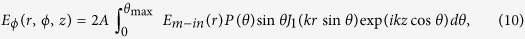


where *E*_*r*_(*r*, *ϕ*, *z*), *E*_*z*_(*r*, *ϕ*, *z*), and *E*_*ϕ*_ (*r*, *ϕ*, *z*) represent the radial, longitudinal, and azimuthal field components, respectively. 
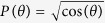
 is the pupil apodization function of the sine condition aplanatic lens, and *θ*_max_ denotes the maximal focusing angle, determined by the NA of the objective lens. Note that the incident field 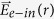 with radial polarization contributes to the radial and longitudinal field components near the focal region, while the azimuthally polarized input field 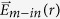 contributes only to the azimuthal field component near the focus^32^.

### Generation of coaxially equidistant multi-focus

**Figure 3:**
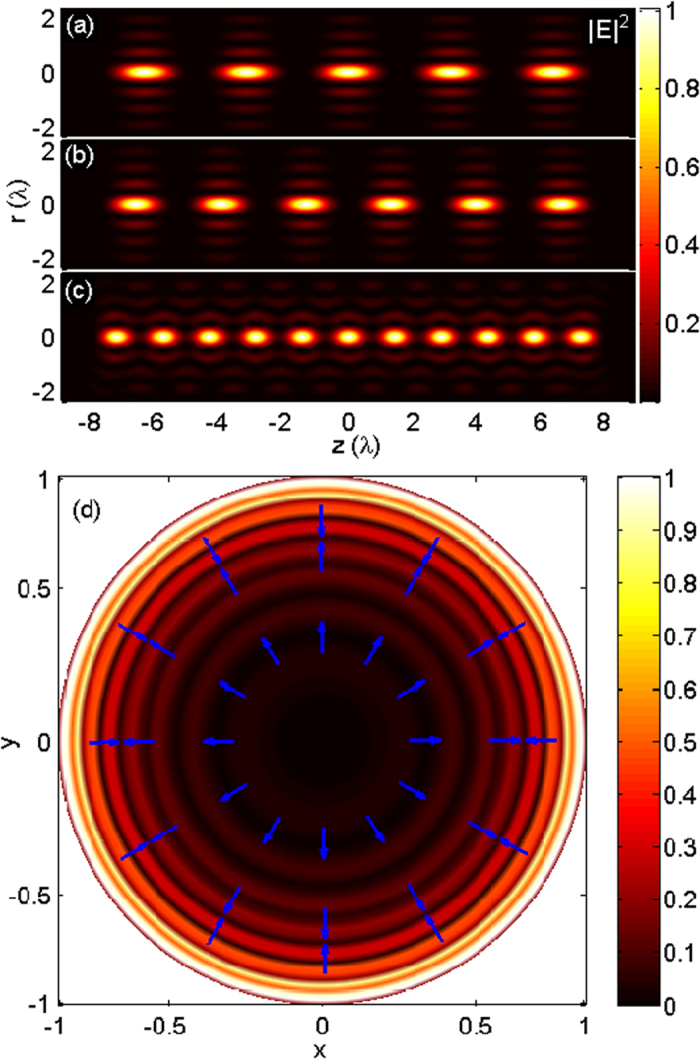
Generation of coaxially equidistant multi-focus using the radiation field of the electric current tapered line source. Contours of |*E*|^2^ in the r–z plane for (**a**) *N* = 5, (**b**) *N* = 6, and (**c**) *N* = 11. (**d**) Required incident field distribution for *N* = 6 at the normalized pupil plane.

To illustrate the focusing behaviors of the proposed method, numerical calculations have been performed for three types of tapered line sources: electric current tapered line source, magnetic current tapered line source, and electromagnetic current tapered line source. Three examples for each case, i.e., for *N* = 5, 6, 11, are presented. The other relevant parameters are taken as *C*_*e*_ = 1, *C*_*m*_ = 1, NA = 1, *θ*_max_ = 90°, *L* = 16λ, and *β*_*0*_ = 0 in all analyses. In the first case, by reversing and focusing the radiation field of an electric current line source with the cosine-squared taper in a 4Pi configuration, the electric field in the vicinity of the focus can be calculated using Eqs. (8) and (9). Figure 3(a–c) illustrate the total electric field intensities, |*E*|^2^ = |*E*_*r*_|^2^ + |*E*_*z*_|^2^, for *N* = 5, 6, 11, respectively, through the geometrical focus of the lenses in the r–z plane. From Eq. (5), the required input field at the pupil plane of the high NA lens for creating these focal patterns can be calculated as a radially polarized field with spatial amplitude modulation, as shown in Fig. 3(d) with *N* = 6. It can be observed from Fig. 3(a–c) that the equidistant uniform multi-focus with five, six, and eleven foci align within the range of −8λ ~ +8λ along the optical axis for *N* = 5, 6, 11, respectively. According to the calculations, higher *N* generally leads to smaller axial spot size, slightly larger radial spot size, and shorter spacing between two adjacent spots. Moreover, the values of the radial component |*E*_*r*_|^2^ are always very small, and the created multiple spots are thus predominantly longitudinally polarized. In fact, the focal pattern with coaxially equidistant multi-focus corresponds to the electric current distribution of the tapered line source. For instance, in Fig. 2, the electric current amplitude has five maximums, and the corresponding focal field has the same number of foci (see Fig. 3(a)). This rule is also applicable to the other two cases that will be discussed in the following text.

### Creation of uniform doughnut spots

**Figure 4:**
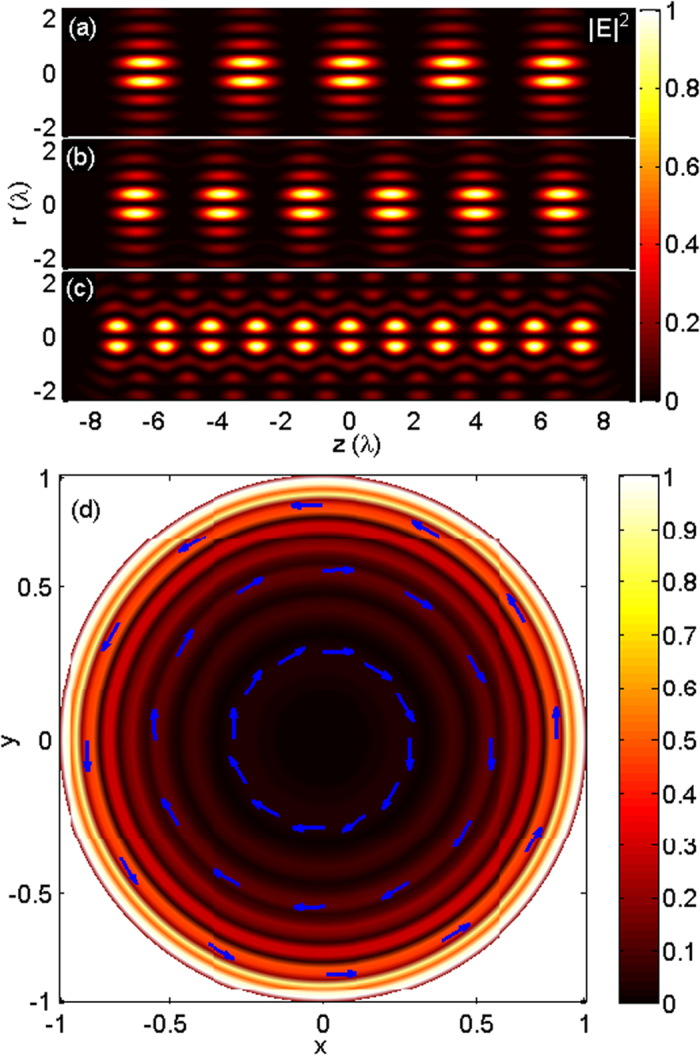
Creation of uniform doughnut spots along the optical axis by focusing the eradiated field from the magnetic current tapered line source. Contours of |*E*|^2^ in the r–z plane for (**a**) *N* = 5, (**b**) *N* = 6, and (**c**) *N* = 11. (**d**) Required pupil field distribution for *N* = 6 at the normalized pupil plane.

Similar to the first case, using Eq. (10), we can compute the focal field distribution generated by the magnetic current tapered line source with *N* = 5, 6, 11, as illustrated in Fig. 4(a–c), respectively. Each focal spot is now a doughnut shape with a dark center surrounded by a high-intensity ring, due to the azimuthally polarized illumination obtained from Eq. (6) (as shown in Fig. 4(d)). The focal fields with 5, 6, and 11 donut-shaped foci are uniformly distributed within the range of −8λ ~ +8λ. The sizes of the doughnut spot along the axial and transverse direction with respect to the parameter *N* show the same changing trend as that of the first case. The phenomena that the focusing of the radiated field from the magnetic current line source exhibits multiple doughnut spots along the optical axis can be understood as follows. It is known that an imaginary magnetic dipole can be regarded as equivalent to a small electric current loop. Therefore, the equivalent of the magnetic current line source with the cosine-squared taper is a series of small current loops, and each loop corresponds to a doughnut spot.

### Production of identical spherical spots

**Figure 5:**
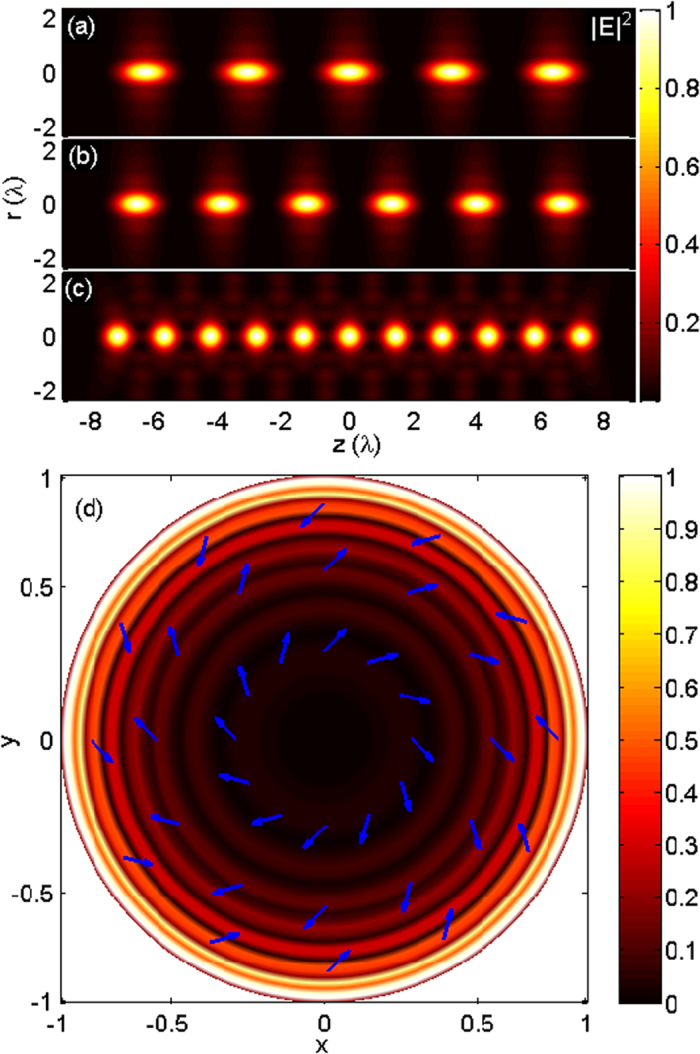
Production of identical spherical spots along the optical axis through revering the radiation pattern from the electromagnetic current tapered line source. Contours of |*E*|^2^ in the r–z plane for (**a**) *N* = 5, (**b**) *N* = 6, and (**c**) *N* = 11. (**d**) Required illumination at the pupil plane for *N* = 6 at the normalized pupil plane.

**Figure 6:**
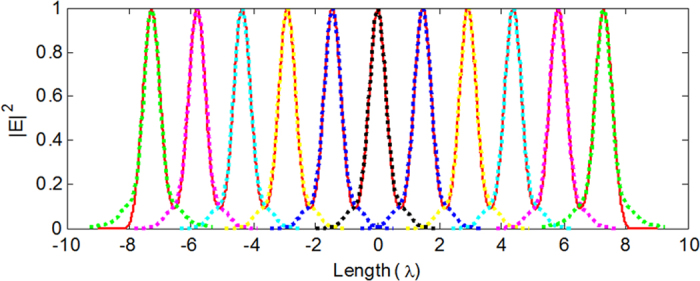
Line scans of corresponding transversal and axial intensity distributions of Fig. 5(c). The dashed lines denote the transversal line scans of 11 spots, and the solid red line denotes the axial line scan.

**Figure 7:**
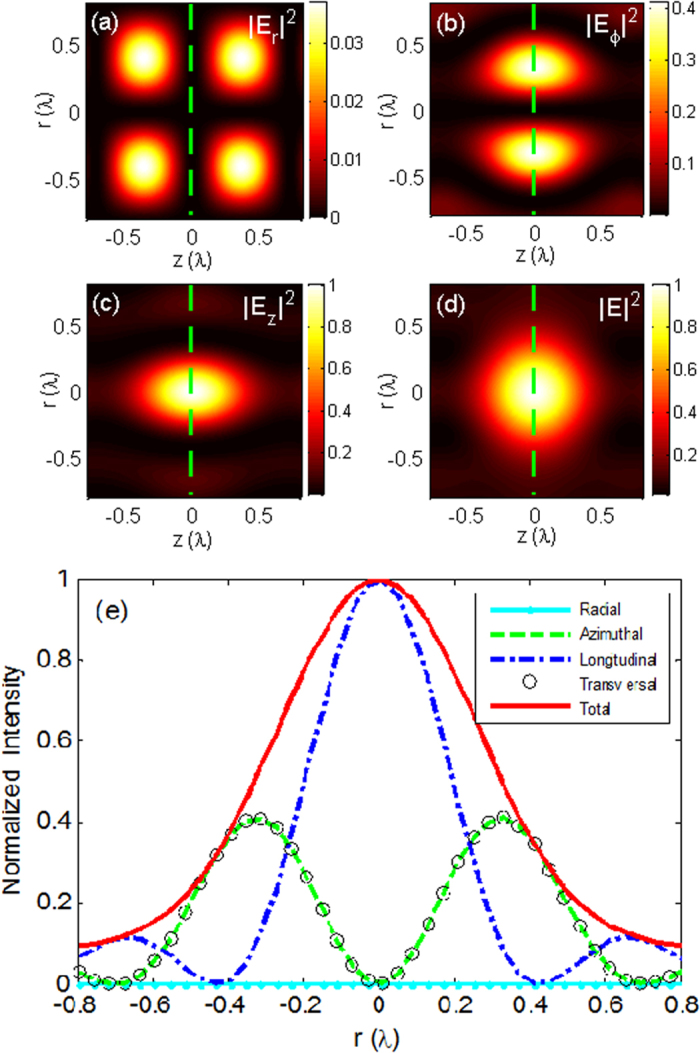
2D contour plots. (**a**) The radial component |*E*_*r*_|^2^, (**b**) the azimuthal component |*E*_*ϕ*_|^2^, (**c**) the longitudinal component |*E*_*z*_|^2^, and (**d**) the total intensity |*E*|^2^ in the r–z plane for the center spot given in Fig. 5(c). Line scans of corresponding intensity distributions in the focal cross section, as marked by the dashed lines in Fig. 7(a–d), are presented in Fig. 7(e).

In the third case, using Eqs. (8)–(10), 13, 14, the identical spherical spots along the optical axis are readily attainable by the use of the radiation fields from the electromagnetic current tapered line sources with parameters *N* = 5, 6, 11, as illustrated in Fig. 5(a–c), respectively. Using Eq. (7), the required illumination for generating these spherical spots can be readily calculated. It is a generalized cylindrical polarization that is spatially modulated in amplitude with eight annular bright belts separated by dark rings, as plotted in Fig. 5(d) for *N* = 6. Each spot is compressed axially and is stretched radially as the value of *N* increases. When *L*/*N* ≈ 1.45λ, focal spots with almost perfect spherical symmetry are achieved. For example, 11 spots with excellent spherical symmetry can be observed in Fig. 5(c). To further demonstrate the symmetry property of the spherical spots plotted in Fig. 5(c), the corresponding line scans of the axial and transversal intensity distributions are shown in Fig. 6. Each spot, with approximately equal axial and transversal focal spot sizes, is formed, and their peak intensities are nearly identical. The 2D contour plots of the radial component |*E*_*r*_|^2^, the azimuthal component |*E*_*ϕ*_|^2^, the longitudinal component |*E*_*z*_|^2^, and the total intensity |*E*|^2^ = |*E*_*r*_|^2^ + |*E*_*ϕ*_|^2^ + |*E*_*z*_|^2^ in the r–z plane for the center spot shown in Fig. 5(c) are illustrated in Fig. 7(a–d), respectively. Line scans of the corresponding intensity distributions in the focal cross section (marked by the dashed lines in Fig. 7(a–d)) are presented in Fig. 7(e). All intensities are normalized to the maximum of the total intensity. The radial component |*E*_*r*_|^2^ has two donut shapes with zero amplitude on the optical axis and contributes very little to the total intensity because of a very low intensity value, especially at the center spot (see Fig. 7(a)). The azimuthal component |*E*_*ϕ*_|^2^ exhibits only a donut pattern, and its maximum intensity is less than half of the peak intensity of the longitudinal component. The spots given in Fig. 5(a–c) have better spherical symmetry than those shown in Fig. 4(a–c) because the azimuthal component |*E*_*ϕ*_|^2^ contributes to the focal spot and causes an elongation of the spot size in the transverse direction. The longitudinally polarized component |*E*_*z*_|^2^ has its peak equal to the maximum of the total intensity on the optical axis and is dominant over the total intensity distribution.

Several conclusions can be drawn from these results: (a) all identical focal spots (including those that are bright and doughnut-shaped) are evenly distributed within the range of –*L*/2 ~ +*L*/2 along the optical axis; (b) the interval between two adjacent spots is approximately *L*/*N*; (c) the number of spots is only determined by the parameter *N*; (d) the required incident field for producing the desired focal spot distribution is spatially modulated in amplitude with 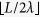 annular bright zones separated by dark rings; and (e) the multiple spots are located symmetrically at the two sides of the geometric focal plane. If *N* is an odd integer number, then a focal spot exists at the geometrical focus. The spot at this location disappears when *N* is even. Note that when *N* ≥ (*L/*λ), the distance between two adjacent spots will be compressed, and as a result, these spots will be combined into a quasi-optical needle or quasi-optical tunnel.

## Conclusion

In summary, we have demonstrated that coaxially equidistant multiple focal spots, including bright spots, doughnut spots, and spherical spots, with a prescribed number and interval can be readily engineered by inversing and focusing the electric fields emitted from the tapered line sources with electric current, magnetic current, and electromagnetic current distributions, respectively, in the 4Pi configuration. The corresponding distributions of the required input field at the pupil plane of the high NA lens for creating these peculiar spots are found to be spatially modulated radial polarization, azimuthal polarization, and generalized cylindrical polarization. Numerical results show that the created multiple spots are distributed symmetrically at the two sides of the original focal plane in the range of −*L*/2 ~ +*L*/2 along the optical axis. The number of focal spots is determined by the parameter *N*, and the distance between two adjacent focal spots is roughly equal to *L*/*N*. The proposed approach for generating identical multiple spots does not require any optimization procedure and therefore is simpler and more flexible than those methods previously reported. The special focal field distributions created with the proposed method may play significant roles in many systems, such as laser direct writing, multiple-particle trapping and manipulation, stimulated emission depletion (STED) microscopy, and optical tweezers.

## Additional Information

**How to cite this article**: Yu, Y. and Zhan, Q. Creation of identical multiple focal spots with prescribed axial distribution. *Sci. Rep.* **5**, 14673; doi: 10.1038/srep14673 (2015).
